# Translation and validation of the Japanese version of the measure of moral distress for healthcare professionals

**DOI:** 10.1186/s12955-021-01765-1

**Published:** 2021-04-13

**Authors:** Tomoko Fujii, Shinshu Katayama, Kikuko Miyazaki, Hiroshi Nashiki, Takehiro Niitsu, Tetsuhiro Takei, Akemi Utsunomiya, Peter Dodek, Ann Hamric, Takeo Nakayama

**Affiliations:** 1 Intensive Care Unit, Jikei University Hospital, 3-25-8, Nishi-Shimbashi, Minato-ku, Tokyo, Japan; 2 Kyoto University Graduate School of Medicine, Yoshida Konoe-cho, Sakyo-ku, Kyoto, Japan; 3 Division of Intensive Care, Department of Anesthesiology and Intensive Care Medicine, Jichi Medical University School of Medicine, 3311-1 Yakushiji, Shimotsuke, Tochigi Japan; 4 Department of Health Informatics, Kyoto University School of Public Health, Yoshida Konoe-cho, Sakyo-ku, Kyoto, Japan; 5 Intensive Care Unit, Iwate Prefectural Central Hospital, 1-4-1 Ueda, Morioka, Iwate Japan; 6 Department of Pediatric Critical Care Medicine, Saitama Children’s Medical Center, 1-2 Shintoshin, Chuo-ku, Saitama, Japan; 7 Intensive Care Unit, Yokohama City Minato Red Cross Hospital, 3-12-1 Shinyamashita, Naka-ku, Yokohama, Kanagawa Japan; 8 Department of Critical Care Nursing, Human Health Sciences, Kyoto University Graduate School of Medicine, 53 Kawaharacho, Shogoin, Sakyo-ku, Kyoto, Japan; 9 Center for Health Evaluation and Outcomes Sciences and Division of Critical Care Medicine, St. Paul’s Hospital and University of British Columbia, Vancouver, BC Canada; 10 Virginia Commonwealth University School of Nursing, E Leigh St, Richmond, VA USA

**Keywords:** Healthcare professional, Moral distress, Japanese, Validation, Factor analysis

## Abstract

**Objectives:**

Moral distress occurs when professionals cannot carry out what they believe to be ethically appropriate actions because of constraints or barriers. We aimed to assess the validity and reliability of the Japanese translation of the Measure of Moral Distress for Healthcare Professionals (MMD-HP).

**Methods:**

We translated the questionnaire into Japanese according to the instructions of EORTC Quality of Life group translation manual. All physicians and nurses who were directly involved in patient care at nine departments of four tertiary hospitals in Japan were invited to a survey to assess the construct validity, reliability and factor structure. Construct validity was assessed with the relation to the intention to leave the clinical position, and internal consistency was assessed with Cronbach’s alpha. Confirmatory factor analysis was conducted.

**Results:**

308 responses were eligible for the analysis. The mean total score of MMD-HP (range, 0–432) was 98.2 (SD, 59.9). The score was higher in those who have or had the intention to leave their clinical role due to moral distress than in those who do not or did not have the intention of leaving (mean 113.7 [SD, 61.3] vs. 86.1 [56.6], t-test *p* < 0.001). The confirmatory factor analysis and Cronbach’s alpha confirmed the validity (chi-square, 661.9; CMIN/df, 2.14; GFI, 0.86; CFI, 0.88; CFI/TLI, 1.02; RMSEA, 0.061 [90%CI, 0.055–0.067]) and reliability (0.91 [95%CI, 0.89–0.92]) of the instrument.

**Conclusions:**

The translated Japanese version of the MMD-HP is a reliable and valid instrument to assess moral distress among physicians and nurses.

**Supplementary Information:**

The online version contains supplementary material available at 10.1186/s12955-021-01765-1.

## Introduction

Moral distress is painful feelings, emotions, and psychological imbalance that occurs when health care professionals (HCPs) cannot perform an action according to their core values or what they believe to be ethically correct [1–3]. The term was first coined in 1984 by Jameton [4]. Initial investigation focused on the situation that nurses are led to do things that they believe to be morally wrong by institutional policies or practices [5]. Moral distress makes HCPs feel compromised and conflicted, leading to burnout and attrition from the workplace [1, 6]. This problem lies in regular clinical practice as HCPs, in particular physicians and nurses, witness life and death through their work.

The theoretical and conceptual model of moral distress have yet to be established [7, 8]; however, research into moral distress has led to the development of tools to measure it. Root cause analysis played an important role to identify the sources of moral distress, and the first scale, which described clinical situations that critical care nurses encountered was created in 2001 [9]. Several revisions to make the scale applicable across various settings and healthcare professionals have been created. The Moral Distress Scale-Revised (MDS-R) was developed based on the following three categories of root causes of moral distress: 1. Clinical situations, e.g. providing unnecessary or futile treatment, 2. Internal constraints, e.g. perceived powerlessness, 3. External constraints, e.g. inadequate communication among team members [10]. Research using the MDS-R revealed that moral distress was associated with age or years of experience of HCPs [1, 11] and the intention to leave a clinical position [1, 10–12]. In 2018, the Measure of Moral Distress for Healthcare Professionals (MMD-HP) was developed based on the MDS-R [3]. The development of MMD-HP was to incorporate recently reported root causes not captured by the MDS-R; e.g. excessive documentation requirements, and administrative directives that diminish the quality of care [10]. The original MMD-HP was developed in English and well-validated in the cohort of healthcare professionals in the United States.

Japan has been experiencing a ‘super-aged’ era where more than 1 in 5 people are aged 65 or older, and increased health care demands for several decades. However, the ratio of the number of hospital beds to practicing physicians was 5.40 in 2016 which is almost five times higher than that in the United States [13]. Japan is known as a country that has some of the longest working hours in the world, which may impose various stresses including moral distress on health care professionals. Moral distress in acute care professionals was one of the key topics at national conferences recently convened by acute care societies in Japan.

Given the dearth of knowledge of moral distress among Japanese healthcare professionals and also the recent awareness of the need to ensure well-being of healthcare professionals, a measure of moral distress that can be used for Japanese healthcare professionals is needed.

### Objectives

This study reports the translation of the MMD-HP into Japanese and the validation of this instrument in the Japanese healthcare professionals.

## Methods

### Questionnaire

The original version of the MMD-HP was developed in English by researchers in the United States [3]. The MMD-HP consists of 27 short situations of potential moral compromise, and each item includes a semi-quantitative score for frequency (e.g., how often the situation is experienced) and for level of distress (e.g., how disturbing the situation is). Responses are given on a five-point Likert scale ranging from 0 (never) to 4 (very frequently) for the frequency scale and from 0 (none) to 4 (great extent) for the level of distress scale. For each item, a composite score is computed by multiplying the frequency and the level of distress scores. The total MMD-HP score is obtained by summing up frequency × distress scores and ranges from 0 to 432. The latest MMD-HP proved to have good internal consistency and validity among US healthcare professionals [3]. Exploratory factor analysis of the MMD-HP revealed four factors: system, clinical/patient, team/personal threat or vulnerability, and team/patient interactions [3].

An open question at the end of the questionnaire allows participants to list any other situations of moral distress that they have experienced. In the questionnaire, moral distress is defined as stress that occurs “when professionals cannot carry out what they believe to be ethically appropriate actions because of constraints or barriers”. In order to test the construct validity, we asked two questions on the intention to leave a clinical position due to the moral distress currently and in the past.

### Translation and pilot testing of MMD-HP

The translation was conducted according to the translation procedure recommended by EORTC [14].

After receiving permission from the developer (AH), forward translation of the MMD-HP was independently performed by two informed translators who were physicians, native Japanese speakers, and fluent in English. A reconciliation of the Japanese translation was made through discussion with a third translator who was a native Japanese speaker and naïve to the outcome measure. Then the reconciled translation was translated back into English by two native-English speaking translators, who were blinded to the original English version. This back-translation was reviewed by the developer of the original MMD-HP, followed by a discussion about problematic items. Then the preliminary translation was sent to the Japanese members of the research group for proofreading. After the wording was agreed upon, the translated MMD-HP was pilot-tested on eight healthcare professionals (three men, five women; two physicians, four nurses, two biomedical equipment technicians who had a mean age of 37 years) to check its comprehensibility in Japanese and the cultural adaptation. Participants of the pilot test were selected from hospitals where the validation study was not conducted to avoid overlap.

### Study settings and participants for validation

The validation study of the Japanese MMD-HP was done in nine departments (emergency department, intensive care unit, general surgery, trauma surgery, thoracic surgery, general internal medicine, hematology, oto-rhino laryngology) of four hospitals, in four prefectures. One hospital was academic, and the other three hospitals were non-academic hospitals. Two hospitals were located in urban areas, and two hospitals were in suburban areas.

The mean capacity of the five hospitals was 682 beds (Standard Deviation [SD], 319). The research ethics committee at Kyoto University and all the four hospitals approved the study (R1239). All 415 physicians and nurses who were directly involved in patient care at the departments were invited to participate.

### Sample size calculation

As there is no determination of sample size related to the psychometric validation with CFA [15], we estimated the required sample size of 270 according to the well-used rule of 10 observations per variable.

### Data collection

The questionnaire was distributed with a cover letter to the participants in December 2017. Completed questionnaires were put in an opaque envelope and returned in boxes located in each department. The survey period was ten days. A reminder was sent at the mid-point of the study period.

### Statistical analysis

We tested two hypotheses to examine the construct validity based on earlier literature on the development of MMD-HP [10]. First, we hypothesized that more experienced healthcare professionals would have higher levels of moral distress [1]. A generalized linear regression analysis was used to test the hypothesis. Second, we hypothesized that healthcare professionals who are or were contemplating leaving their clinical position due to moral distress would have higher scores [10]. Student’s t-test was used to test the second hypothesis.

Furthermore, to verify the internal structure of the instrument that was observed in the original MMD-HP, we performed a confirmatory factor analysis (CFA) of the 27 items. Estimates were calculated by the maximum likelihood method. To assess the goodness of fit, chi-square, chi-square to degrees of freedom ratio (CMIN/df, < 3.0 for good model fit), goodness of fit index (GFI, ≥ 0.90), comparative fit index (CFI, ≥ 0.90) and CFI to Tucker Lewis index (TLI) ratio (CFI/TLI, ≥ 0.95), the root mean square error of approximation (RMSEA, < 0.08) were used. Scale reliability was assessed by measuring internal consistency using Cronbach’s alpha [16]. A minimum value of 0.70 is generally desirable.

In addition, we conducted an exploratory factor analysis (EFA) to extract factors specific to the Japanese cohort. The additional analysis was done to explore a possible factor structure that can be identified within the original 27 items so as to elaborate on the MMD-HP domestically and internationally. Using parallel analysis, we identified the number of factors and factor analysis with Promax rotation was conducted to allow factor intercorrelations. Ad-hoc analysis for the number of factors was conducted using the revised Velicer's minimum average partial (MAP) Test [17]. We performed exploratory CFA for the reconstructed factor structure to confirm the suggested structure in the EFA.

Analyses were conducted using R version 3.4.1 (2017-06-30) for EFA, and IBM SPSS Amos 25.0.0 (Wexford, PA, USA) for CFA. A two-tailed *p* value < 0.05 was considered to be statistically significant.

All those involved in any aspect of the study was mentioned above and in the Acknowledgement. Patients and the public were not involved in this study.

## Results

**Fig. 1:**
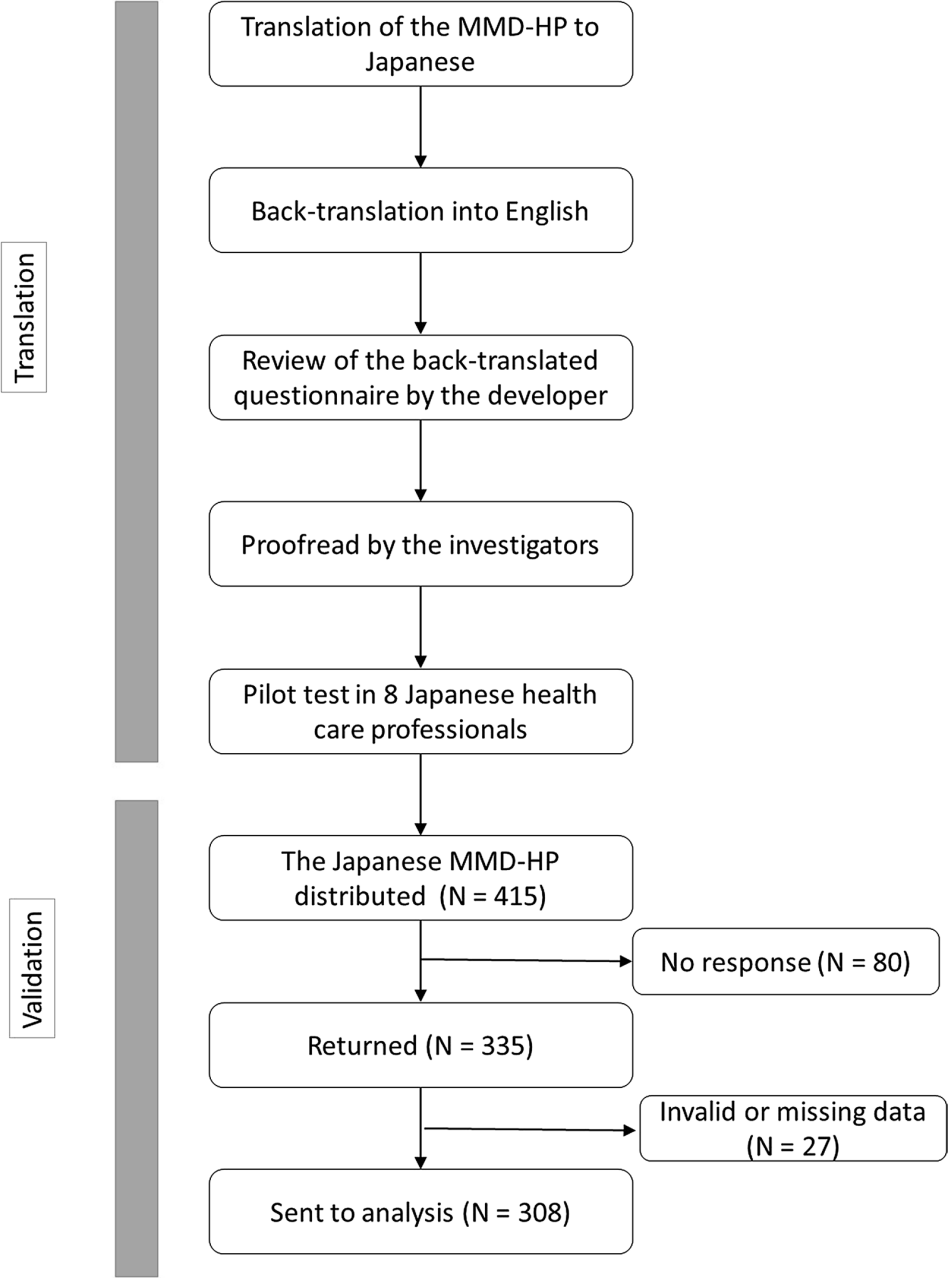
Study flow

The study flow is presented in Fig. 1.

### Translation

The word “care” in English, which means “ケア” in Japanese seemed not suitable as the word “ケア” suggests nursing care in Japanese context and was translated to “診療/ケア” (clinical practice/care). The back-translated version of the Japanese MMD-HP was similar to the original MMD-HP and adjudicated to be acceptable by the original developer (AH). In the pilot test, the Japanese MMD-HP was understood well by selected physicians, nurses and other healthcare professionals who are native Japanese users.

### Respondents demographic

We distributed 415 questionnaires, and 335 (81%) were returned. Twenty-seven of 335 responses were excluded because at least one items in the questionnaire to calculate MMD-HP score were unanswered: thus, 308 (74%) were included into the analysis. The response rate was 71.3% for physicians and 83.2% for nurses. The mean age of the participants was the early 30 s, and nurses who participated in the study were five years younger than the physicians on average (Table 1). More than 70 percent of the participants were female.

**Table 1:** Participants characteristics and the MMD-HP score

	Overall	N = 308	Physicians	N = 57^a^	Nurses	N = 242^a^	p-value^b^
Age, years	32.6	(8.4)	36.3	(7.7)	31.7	(8.4)	< 0.001
Male, female	85 (27.6)	223 (72.4)	48 (84.0)	9 (16.0)	30 (12.0)	212 (88.0)	< 0.001
Department/Ward							0.241
Medical	73	(24.5)	18	(31.6)	55	(23.0)	
Surgical	98	(32.9)	14	(24.6)	83	(34.7)	
Critical/Emergency	127	(42.6)	25	(43.9)	101	(42.3)	
Clinical experiences, years	8.9	(7.4)	10.0	(5.9)	8.6	(7.6)	0.276
MMD-HP score	98.2	(59.9)	104.0	(63.4)	97.2	(59.3)	0.444

^a^9 participants did not answer their occupation

^b^*p*-value of the test for the difference between physicians and nurses

### MMD-HP score

**Fig. 2:**
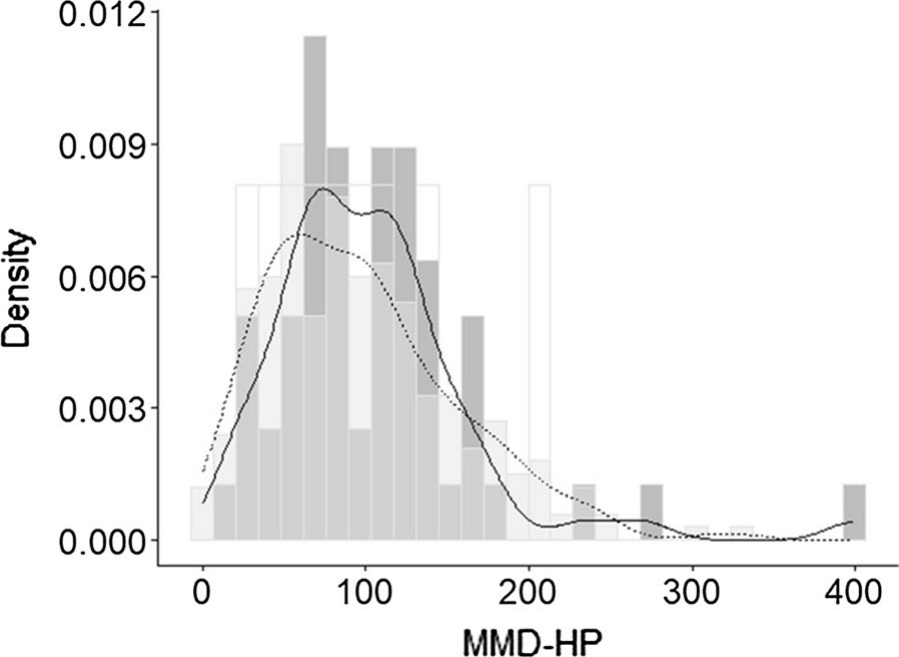
Distribution of the MMD-HP score and kernel density curves stratified by the occupation. Dark gray bar and a solid line = physicians, light gray bar and a dotted line = nurses, white bar = unknown

The mean total score of MMD-HP was 98 (SD, 60) and the difference of the score between physicians and nurses was not statistically significant (104 [63] v.s. 97 [59], *p* = 0.4, Fig. 2). The 10 top-ranked items are shown in Table 2. Eight out of the 10 items were the same between physicians and nurses. Participant characteristics and the MMD-HP scores by gender or department are reported in Additional file 1: Table 1.

**Table 2:** Top 10 ranked items in the MMD-HP questionnaire by occupations

Rank	Physicians			Nurses			
	Item	Mean	(SD)	Item		Mean	(SD)
1	Follow the family’s insistence to continue aggressive treatment even though I believe it is not in the best interest of the patient	8.2	(4.6)	Follow the family’s insistence to continue aggressive treatment even though I believe it is not in the best interest of the patient		7.7	(4.3)
2	Feel pressured to order or carry out orders for what I consider to be unnecessary or inappropriate tests and treatments	6.3	(4.5)	Be required to care for more patients than I can safely care for		6.3	(5.3)
3	Be required to care for more patients than I can safely care for	6.3	(6.0)	Participate in care that causes unnecessary suffering or does not adequately relieve pain or symptoms		5.0	(4.4)
4	Witness low quality of patient care due to poor team communication	5.7	(4.4)	Feel pressured to order or carry out orders for what I consider to be unnecessary or inappropriate tests and treatments		4.9	(4.1)
5	Be required to care for patients who have unclear or inconsistent treatment plans or who lack goals of care	5.4	(4.7)	Be required to care for patients who have unclear or inconsistent treatment plans or who lack goals of care		4.9	(4.5)
6	Have excessive documentation requirements that compromise patient care	5.3	(5.2)	Witness low quality of patient care due to poor team communication		4.8	(4.7)
7	Watch patient care suffer because of a lack of provider continuity	5.3	(4.8)	Be required to work with abusive patients/family members who are compromising quality of care		4.5	(4.8)
8	Be required to work with other healthcare team members who are not as competent as patient care requires	5.1	(4.8)	Be required to work with other healthcare team members who are not as competent as patient care requires		4.5	(4.7)
9	Be required to work with abusive patients/family members who are compromising quality of care	5.0	(5.0)	Have excessive documentation requirements that compromise patient care		4.4	(5.0)
10	Witness healthcare providers giving “false hope” to a patient or family	4.9	(4.6)	Continue to provide aggressive treatment for a person who is most likely to die regardless of this treatment when no one will make a decision to withdraw it		4.4	(4.3)

### Hypothesis testing to confirm convergent validity

By regression analysis using a generalized linear model, the length of clinical experience was weakly but positively associated with the MMD-HP score (β 0.16, 95%CI, 0.08 to 0.24, *p* < 0.001), which supported the predefined hypothesis that the experienced healthcare professionals would have higher levels of moral distress. The MMD-HP score was higher in those who had the intention to leave their clinical role due to moral distress than in those who did not have the intention of leaving (mean 120 [SD, 60] v.s. 84 [57], t-test *p* < 0.001). And the MMD-HP score was higher in those who currently have the intention to leave their clinical role due to moral distress than in those who do not, but the difference was not statistically significant (mean 113 [SD, 54] v.s. 96 [62], t-test *p* = 0.075). When we combined the past and current intention to leave the position, the score was higher if a participant had or has the intention to leave the position (mean 114 [SD, 61] v.s. 86 [57], t-test *p* < 0.001).

### Confirmatory factor analysis

**Fig. 3:**
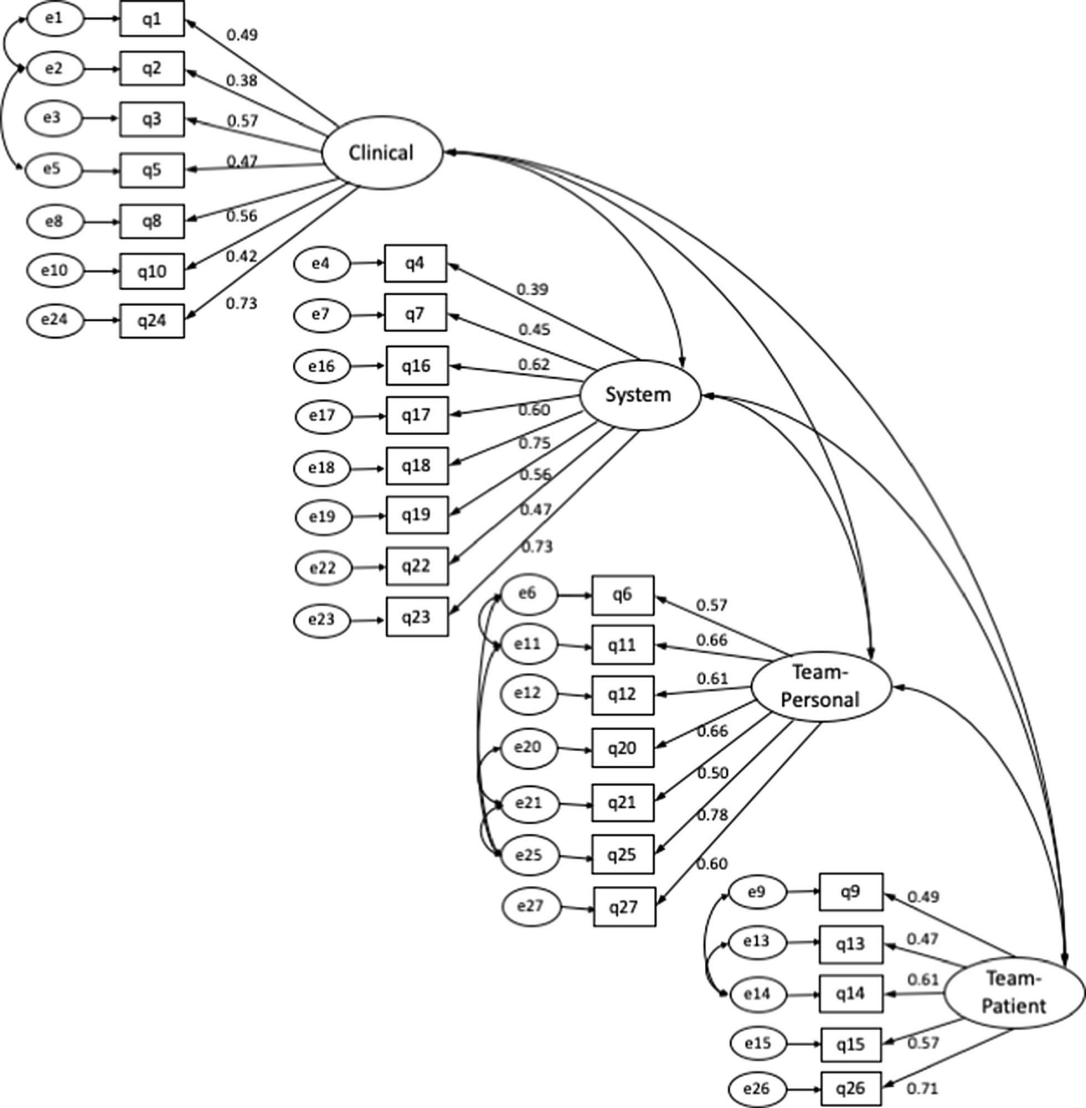
Path diagram of the confirmatory factor analysis with the standardized coefficients of the Japanese MMD-HP

We conducted CFA to validate the four-factor structure proposed by the original developer (Fig. 3). The chi-square of the model was 661.9 and CMIN/df of the model was 2.14. The GFI was 0.86. The CFI was 0.88 and the CFI/TLI was 1.02. The RMSEA of the model was 0.061 (90% CI, 0.055 to 0.067). Some moral distress root causes operate at multiple levels. The validation study of the original MMD-HP reported Q22 to be loaded by two factors, and we further we reviewed the items to identify Q13, Q24 to be possibly attributable to two factors. However, model fit was not significantly changed when we assumed three items (Q13, Q22, Q24) were loaded from two factors (Additional file 1: Fig. 1).

### Internal consistency

The Cronbach’s alpha of the Japanese MMD-HP was 0.91 (95%CI, 0.89 to 0.92).

### Additional Exploratory and confirmatory factor analysis in the Japanese cohort

Parallel analysis showed the number of factors in the Japanese cohort was three (Additional file 1: Fig. 2). The ad-hoc analysis using Velicer’s minimum average partial test suggested a single factor (Additional file 1: Fig. 3). We conducted EFA using all the 27 items in the original MMD-HP and interpreted the three factors as causes attributed to clinical situations, system, and team. The EFA showed that five items (Q4, Q7, Q9, Q13, and Q22) had poor factor loadings (< 0.32, Additional file 1: Table 2). The result of EFA rerun after deleting the five items is shown in Table 3. The variance explained by the three factors was 38.2%. After excluding these five items, CFA was conducted to validate the three-factor structure with 22 items (Additional file 1: Fig. 4). The chi-square of the model was 418.9 and the CMIN/df was 2.16. The GFI was 0.891. The CFI was 0.91 and the CFI/TLI was 1.02. The RMSEA of the model was 0.061 (90% CI, 0.053 to 0.070). The ad-hoc EFA with 1-factor, 2-factor, 4-factor, 5-factor and 6-factor structure and their factor loadings are reported in Additional file 1: Table 3).

**Table 3:** Factor structure matrix—Promax rotation. *h*^2^ = communality coefficient

Item	Factor 1	Factor 2	Factor 3	*h*^*2*^
2	**0.74**	−0.02	−0.23	0.396
5	**0.64**	0.03	−0.09	0.377
1	**0.51**	0.07	−0.03	0.290
3	**0.43**	0.12	0.09	0.315
8	**0.41**	0.02	0.19	0.305
10	**0.37**	−0.06	0.16	0.200
24	**0.34**	0.22	0.21	0.438
16	0.05	**0.74**	−0.15	0.462
18	−0.02	**0.70**	0.10	0.589
17	0.09	**0.64**	−0.09	0.404
19	0	**0.60**	−0.03	0.336
23	0.04	**0.46**	0.27	0.482
12	0.10	−0.24	**0.75**	0.459
25	−0.21	0.20	**0.73**	0.590
27	−0.02	−0.03	**0.66**	0.398
6	0	−0.11	**0.65**	0.338
21	−0.11	0.01	**0.60**	0.301
20	−0.23	0.31	**0.58**	0.491
15	0.23	−0.10	**0.53**	0.383
26	0.05	0.21	**0.53**	0.513
11	0.25	0.01	**0.46**	0.417
14	0.01	0.23	**0.41**	0.359

Bold numbers indicated loadings greater than 0.3 and included in the interpretation of factors

## Discussion

### The aim of the study

The aim of the current study was to translate the MMD-HP into Japanese and validate the translated instrument to assess whether it could be used in the Japanese HCPs.

## Summary of key findings from the results

This study examined the psychometric properties of the Japanese version of MMD-HP based on convenient sampling from various departments in Japanese hospitals. There were very few missing values which assure the high quality of the data and results. Overall the performance of the instrument was fair to satisfactory, which was supported by the confirmed predefined hypothesis about the relationship between experience and moral distress, the high internal consistency, and the acceptable model fit.

### Explanations of the findings based on prior studies and the original MMD-HP

Initial studies about moral distress identified three major categories of causes: clinical situations, internal constraints, and external constraints [10]. As more was learned about this issue, there was a recognition that there were other dimensions to be measured. A recent report of a moral distress consultation service revealed that most episodes of moral distress had multiple root causes [18]. These authors identified 32 different root causes which were sorted into three categories: patient/family, unit/team, and organization/system [18]. The MMD-HP was developed to capture all three categories. The exploratory findings of EFA and CFA with three-factor structure in Japanese cohort suggested the different models could be explored. In the development of original MMD-HP, four factors were identified and validated. In the four-factor structure, the team-level factor was split into a cause related to compromises to individual integrity within a team and a cause related to interactions between a team and patients or families. CFA of the four-factor structure confirmed the acceptable model fit in the Japanese cohort as well. Considering that the MMD-HP designated its total score to measure moral distress that sub-scores were not developed nor validated, and also the model fit was similar in the three-factor structure and the four-factor structure, the three-factor structure with 22 items will not supersede the original four-factor structure with 27 items. When researchers aim to compare moral distress between other settings where the original MMD-HP questionnaire is validated and a Japanese population, the four-factor structure measured with 27 items would be useful.

### Strengths of the study

There are several strengths of this study. First, we conducted this validation study of the Japanese version immediately after the development of the MMD-HP in English. This enables multinational, multicultural surveys of moral distress using the newest instrument among English and Japanese users. Second, the process of translation and cross-cultural adaptation was rigorously conducted in line with the recommendation of EORTC [14]. Third, we have included both physicians and nurses, adult and pediatric departments, academic hospitals and non-academic hospitals, and urban areas and suburban areas. These considerations in the recruitment made the findings of this study generalizable. Of note, we have included not only healthcare professionals in the ICU, where the early instruments were focused but also professionals who work outside of ICUs, as the scope of the current instrument has been developed for use beyond the ICU.

### Limitations and implications for future studies

This study has several limitations. First, we recruited hospitals and departments to conduct the survey at the researcher’s convenience. Of note, our study was performed only at tertiary care hospitals. Broader validation in different settings would strengthen the findings in the psychometric properties of the MMD-HP. Second, there was possibly a selection bias because we did not include physicians or nurses who had already left their clinical position due to high moral distress. A highly distressed subgroup in our cohort might be rather modestly distressed than those people who had already left, so that they could continue to work. The survival bias might have led to the weak association of the years of clinical experience and the MMD-HP score and have underestimated the association. Third, we did not examine the validity of the instrument by a criterion-oriented validation. The Hospital Ethical Climate Scale was used in the validation study of the original version [3] but there is no Japanese version of that scale. Fourth, the model fit was not perfectly desirable in CFA for the originally proposed model in the Japanese cohort. Most of the metrics (CMIN/df, CFI/TLI, RMSEA) in CFA confirmed that the model fit well, but the others (GFI, CFI) were not sufficient for the ideal model fit. Also, in the three-structure model in EFA, the proportion of the variance explained by the model was not high. Thus, there may be some unmeasured causes of the moral distress in the Japanese population, which should be further studied in future research. Fifth, we performed additional EFA and CFA in the same dataset. Thus, the additional analysis to explore the factor structures in the Japanese cohort should be taken as exploratory as the model would be overfitted. The three-factor structure suggested by parallel test was not detected in MAP test which suggested only one factor, possibly because the factor loadings were very low in some items. Also, the low variances of the models with various numbers of factors may be explained by the nature of the translated version of a scale. As the questionnaire was developed in the US, where the culture, clinical context, and expressions would differ from those in Japan, the translated questionnaire might not be sufficiently comprehensive. These findings should be explored and examined in a future study which aims to develop a new or revised scale for the Japanese population. Finally, we could not obtain any demographic data on non-responders as the survey was conducted in a way that the participants could not be identified or traced. The anonymization was required to assure the participants’ psychological safety in answering questions that are highly sensitive, particularly because the questionnaire was distributed at their workplace.

## Conclusions

We translated the MMD-HP, which was originally developed in English into Japanese. The translated Japanese version of the MMD-HP is a reliable and valid instrument to assess moral distress among physicians and nurses.

## Supplementary Information

**Additional file 1.** **Supplementary Figure 1.** Assuming 3 items were loaded from 2 factors as in the original exploratory factor analysis. **Supplementary Figure 2.** Scree plot and parallel analysis. **Supplementary Figure 3**. The Velicer’s very simple structure. The Velicer’s MAP criterion achieved a minimum of 0.02 with 1 factor. **Supplementary Figure 4.** Path diagram of the confirmatory factor analysis with 3-factor structure model. **Supplementary Table 1.** Participant characteristics and MMD-HP score by gender and departments. **Supplementary Table 2.** The whole factor structure matrix – Promax rotation. **Supplementary Table 3.** Ad-hoc exploratory factor analysis with various numbers of factors – Promax rotation.

## Data Availability

The datasets during and/or analysed during the current study available from the corresponding author on reasonable request.

## References

[1] Dodek PM, Wong H, Norena M (2016). Moral distress in intensive care unit professionals is associated with profession, age, and years of experience. J Crit Care.

[2] Hamric AB, Blackhall LJ (2007). Nurse-physician perspectives on the care of dying patients in intensive care units: collaboration, moral distress, and ethical climate. Crit Care Med.

[3] Epstein EG, Whitehead PB, Prompahakul C, Thacker LR, Hamric AB (2019). Enhancing understanding of moral distress: the measure of moral distress for health care professionals. AJOB Empir Bioeth.

[4] Jameton A. Nursing Practice: The Ethical Issues. illustrate. Prentice-Hall; 1984. 331 p.

[5] Wilkinson J (1988). Signs of moral distress in nursing practice. Nurs Forum.

[6] Fumis RRL, Junqueira Amarante GA, de Fátima NA, Vieira Junior JM (2017). Moral distress and its contribution to the development of burnout syndrome among critical care providers. Ann Intensive Care.

[7] Pauly BM, Varcoe C, Storch J (2012). Framing the issues: moral distress in health care. HEC Forum.

[8] Barlem EL, Ramos FR (2015). Constructing a theoretical model of moral distress. Nurs Ethics.

[9] Corley MC, Elswick RK, Gorman M, Clor T (2001). Development and evaluation of a moral distress scale. J Adv Nurs.

[10] Hamric AB, Borchers CT, Epstein EG (2012). Development and testing of an instrument to measure moral distress in healthcare professionals. AJOB Prim Res.

[11] Hiler CA, Hickman RL, Reimer AP, Wilson K (2018). Predictors of moral distress in a US sample of critical care nurses. Am J Crit Care.

[12] Trautmann J, Epstein E, Rovnyak V, Snyder A (2015). Relationships among moral distress, level of practice independence, and intent to leave of nurse practitioners in emergency departments: results from a national survey. Adv Emerg Nurs J.

[13] Health Care Resources. Organisation for Economic Co-operation and Development. 2019. Available from: https://stats.oecd.org/index.aspx?DataSetCode=HEALTH_REAC#. Accessed 13 Nov 2019.

[14] Kuli D, Bottomley A, Velikova G, Greimel E. EORTC Quality of Life Group Translation Procedure. 4th ed. 4th ed. EORTC. EORTC; 2017. 1–26 p.

[15] Hudziak J, Achenbach T, Althoff R, Pine D. A dimensional approach to developmental psychopathology. Int J Methods Psychiatr Res [Internet]. 2007;16(S1):S16–23. Available from: http://www.scopus.com/inward/record.url?eid=2-s2.0-79951999327&partnerID=tZOtx3y1 10.1002/mpr.217 PMC6879082 17623391

[16] Cronbach LJ, Meehl PE (1955). Construct validity in psychological tests. Psychol Bull.

[17] O'Connor BP (2000). SPSS and SAS programs for determining the number of components using parallel analysis and Velicer's MAP test. Behav Res Methods Instrum Comput.

[18] Hamric AB, Epstein EG (2017). A health system-wide moral distress consultation service: development and evaluation. HEC Forum.

