# Supplementary material for: Translation and validation of the Japanese version of the measure of moral distress for healthcare professionals
Source: Health Qual Life Outcomes. 2021 Apr 13;19:120. doi: 10.1186/s12955-021-01765-1 (PMC8045393; doi:10.1186/s12955-021-01765-1)
Supplement: Supplementary file 1 — Additional file 1. Supplementary Figure 1. Assuming 3 items were loaded from 2 factors as in the original exploratory factor analysis. Supplementary Figure 2. Scree plot and parallel analysis. Supplementary Figure 3. The Velicer’s very simple structure. The Velicer’s MAP criterion achieved a minimum of 0.02 with 1 factor. Supplementary Figure 4. Path diagram of the confirmatory factor analysis with 3-factor structure model. Supplementary Table 1. Participant characteristics and MMD-HP score by gender and departments. Supplementary Table 2. The whole factor structure matrix – Promax rotation. Supplementary Table 3. Ad-hoc exploratory factor analysis with various numbers of factors – Promax rotation. [file 12955_2021_1765_MOESM1_ESM.docx]

**Supplementary Figure 1**. Assuming 3 items were loaded from 2 factors as in the original exploratory factor analysis.


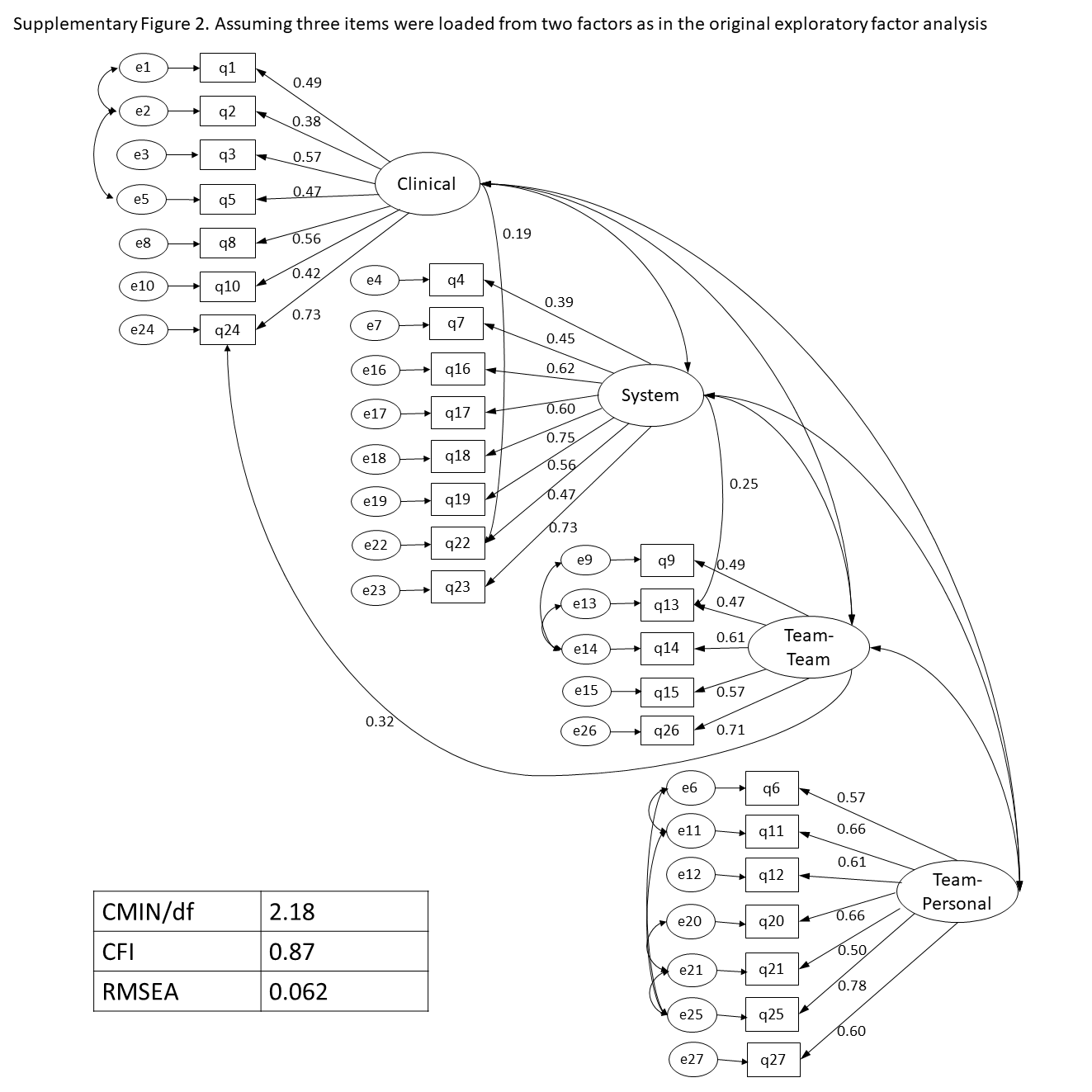


**Supplementary Figure 2**. Scree plot and parallel analysis


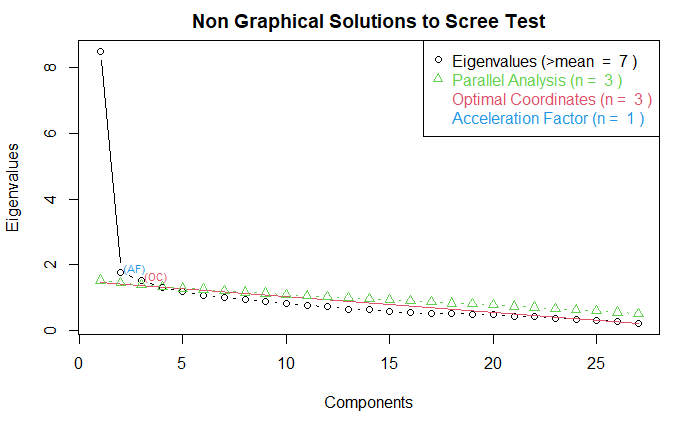


**Supplementary Figure 3**. The Velicer’s very simple structure. The Velicer’s MAP criterion achieved a minimum of 0.02 with 1 factor.


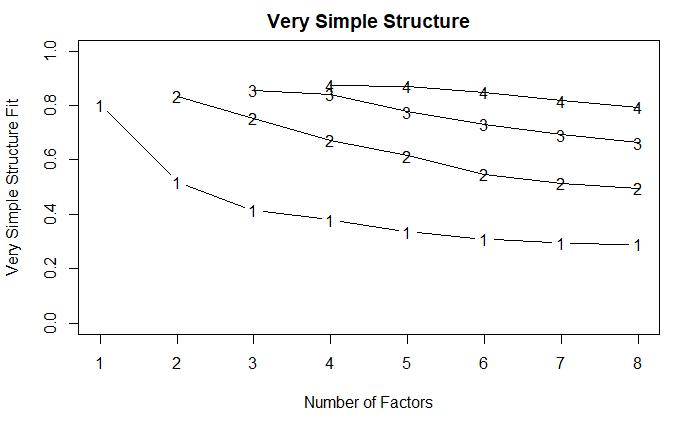


**Supplementary Figure 4**. Path diagram of the confirmatory factor analysis with 3-factor structure model.


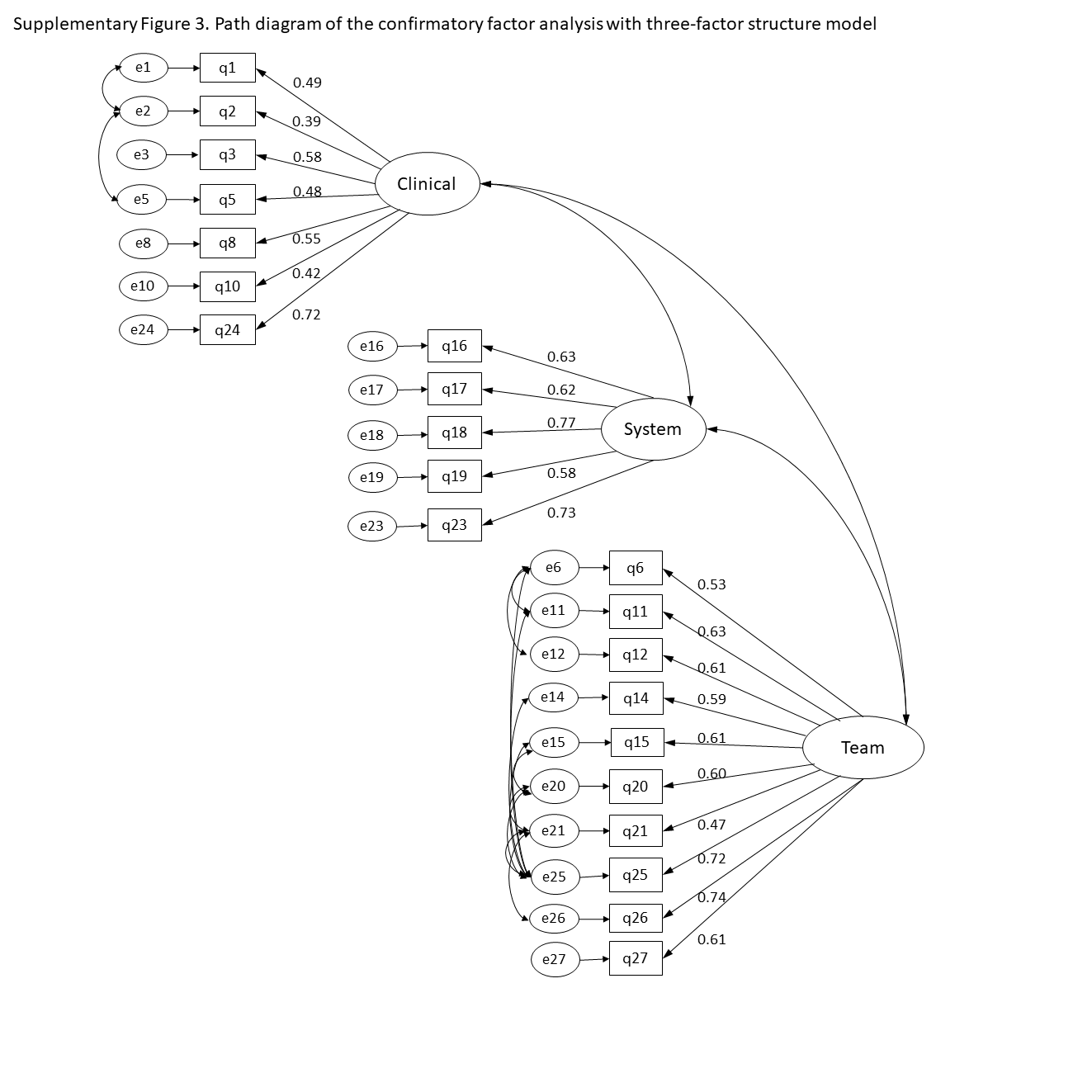


**Supplementary Table 1.** Participant characteristics and MMD-HP score by gender and departments.

a. by gender

|  | Male (N = 76) | Female (N = 223) | p-value |
| --- | --- | --- | --- |
| Age, years | 36.1 (8.1) | 31.4 (8.2) | <0.001 |
| Nurses (%) | 28 (36.8) | 212 (95.9) | <0.001 |
| Clinical experiences, years | 123.2 (81.5) | 102.1 (90.4) | 0.116 |
| MMD-HP score | 103.9 (61.1) | 97.0 (60.3) | 0.385 |

b. by department

|  | Medical (N = 73) | Surgical (N = 98) | Critical/Emergency (N = 127) | p-value |
| --- | --- | --- | --- | --- |
| Age, years | 32.7 (7.7) | 31.6 (9.2) | 33.3 (8.2) | 0.336 |
| Female (%) | 54 ( 74.0) | 78 ( 81.2) | 89 ( 70.1) | 0.157 |
| Nurse (%) | 55 ( 75.3) | 83 ( 85.6) | 101 ( 80.2) | 0.231 |
| Clinical experience, years | 105.4 (81.1) | 93.6 (99.6) | 118.8 (82.5) | 0.158 |
| MMD-HP score | 93.9 (52.6) | 95.6 (66.0) | 104.1 (60.2) | 0.421 |

**Supplementary Table 2.** The whole factor structure matrix – Promax rotation.

| Item | Factor 1 | Factor 2 | Factor 3 |
| --- | --- | --- | --- |
| 2 | **0.74** | -0.05 | -0.24 |
| 5 | **0.68** | 0.01 | -0.12 |
| 1 | **0.52** | 0.04 | -0.04 |
| 3 | **0.47** | 0.13 | 0.03 |
| 8 | **0.44** | 0 | 0.17 |
| 10 | **0.39** | -0.08 | 0.14 |
| 24 | **0.35** | 0.21 | 0.19 |
| 16 | 0 | **0.81** | -0.18 |
| 18 | -0.06 | **0.72** | 0.11 |
| 17 | 0.07 | **0.69** | -0.14 |
| 19 | -0.04 | **0.62** | -0.04 |
| 23 | 0.01 | **0.46** | 0.28 |
| 25 | -0.26 | 0.2 | **0.77** |
| 12 | 0.14 | -0.24 | **0.72** |
| 21 | -0.16 | -0.03 | **0.67** |
| 27 | -0.02 | -0.04 | **0.67** |
| 6 | 0 | -0.14 | **0.66** |
| 20 | -0.29 | 0.3 | **0.63** |
| 15 | 0.24 | -0.14 | **0.53** |
| 26 | 0.03 | 0.2 | **0.53** |
| 11 | 0.26 | -0.02 | **0.47** |
| 14 | 0.03 | 0.24 | **0.41** |
| 13 | 0.22 | 0.26 | 0.11 |
| 4 | 0.21 | 0.24 | 0 |
| 7 | 0.08 | 0.21 | 0.22 |
| 9 | 0.27 | 0.05 | 0.27 |
| 22 | 0.16 | 0.16 | 0.22 |

**Supplementary Table 3.** Ad-hoc exploratory factor analysis with various numbers of factors – Promax rotation.

a. 1-factor structure factor loadings, cumulative variance 29.0%

| q1 | 0.410 |
| --- | --- |
| q2 | 0.319 |
| q3 | 0.517 |
| q4 | 0.386 |
| q5 | 0.430 |
| q6 | 0.493 |
| q7 | 0.461 |
| q8 | 0.515 |
| q9 | 0.519 |
| q10 | 0.376 |
| q11 | 0.627 |
| q12 | 0.57 |
| q13 | 0.500 |
| q14 | 0.619 |
| q15 | 0.565 |
| q16 | 0.522 |
| q17 | 0.524 |
| q18 | 0.669 |
| q19 | 0.469 |
| q20 | 0.612 |
| q21 | 0.464 |
| q22 | 0.481 |
| q23 | 0.67 |
| q24 | 0.645 |
| q25 | 0.671 |
| q26 | 0.694 |
| q27 | 0.575 |

b. 2-factor structure factor loadings, cumulative variance 32.2%

|  | factor1 | factor2 |
| --- | --- | --- |
| q1 |  | 0.556 |
| q2 | -0.336 | 0.758 |
| q3 |  | 0.538 |
| q4 | 0.127 | 0.305 |
| q5 | -0.183 | 0.716 |
| q6 | 0.565 |  |
| q7 | 0.343 | 0.149 |
| q8 | 0.129 | 0.456 |
| q9 | 0.276 | 0.292 |
| q10 |  | 0.374 |
| q11 | 0.426 | 0.249 |
| q12 | 0.545 |  |
| q13 | 0.240 | 0.311 |
| q14 | 0.550 | 0.101 |
| q15 | 0.420 | 0.184 |
| q16 | 0.304 | 0.264 |
| q17 | 0.272 | 0.302 |
| q18 | 0.529 | 0.182 |
| q19 | 0.337 | 0.165 |
| q20 | 0.847 | -0.230 |
| q21 | 0.664 | -0.202 |
| q22 | 0.305 | 0.216 |
| q23 | 0.554 | 0.155 |
| q24 | 0.287 | 0.428 |
| q25 | 0.924 | -0.244 |
| q26 | 0.646 |  |
| q27 | 0.648 |  |

c. 4-factor structure factor loadings, cumulative variance 41.1%

|  | factor1 | factor3 | factor2 | factor4 |
| --- | --- | --- | --- | --- |
| q1 |  |  | 0.444 |  |
| q2 | -0.216 | -0.170 | 0.907 |  |
| q3 |  | 0.131 | 0.428 |  |
| q4 | 0.135 | 0.308 |  |  |
| q5 |  |  | 0.701 |  |
| q6 | 0.800 | -0.105 | -0.249 |  |
| q7 | 0.184 | 0.240 |  |  |
| q8 | 0.497 |  | 0.166 | -0.166 |
| q9 | 0.335 |  | 0.137 |  |
| q10 | 0.520 |  | 0.112 | -0.215 |
| q11 | 0.552 |  |  |  |
| q12 | 0.773 | -0.213 |  | 0.133 |
| q13 | 0.126 | 0.305 | 0.128 |  |
| q14 | 0.198 | 0.263 |  | 0.239 |
| q15 | 0.739 | -0.106 |  |  |
| q16 | -0.212 | 0.882 |  |  |
| q17 |  | 0.814 |  | -0.154 |
| q18 |  | 0.777 |  | 0.114 |
| q19 | -0.154 | 0.654 |  |  |
| q20 |  | 0.219 |  | 0.677 |
| q21 |  | -0.255 | 0.193 | 0.835 |
| q22 | 0.203 | 0.180 |  |  |
| q23 | 0.258 | 0.531 | -0.117 |  |
| q24 | 0.235 | 0.250 | 0.229 |  |
| q25 | 0.103 | 0.120 |  | 0.691 |
| q26 | 0.498 | 0.262 | -0.126 | 0.112 |
| q27 | 0.519 |  |  | 0.260 |

d. 5-factor structure factor loadings, cumulative variance 39.9%

|  | factor1 | factor5 | factor3 | factor4 | factor2 |
| --- | --- | --- | --- | --- | --- |
| q1 |  | 0.112 |  |  | 0.378 |
| q2 | -0.109 |  |  | 0.139 | 0.856 |
| q3 | -0.120 | 0.583 |  |  | 0.292 |
| q4 |  | 0.311 | 0.155 | -0.119 |  |
| q5 |  | 0.134 |  |  | 0.585 |
| q6 | 0.760 |  |  |  | -0.182 |
| q7 | 0.100 | 0.269 | 0.128 |  |  |
| q8 | 0.410 | 0.176 |  | -0.145 | 0.134 |
| q9 | 0.175 | 0.433 |  |  |  |
| q10 | 0.552 | -0.142 |  | -0.156 | 0.145 |
| q11 | 0.543 |  |  |  | 0.102 |
| q12 | 0.563 | 0.370 | -0.287 |  | -0.111 |
| q13 |  | 0.598 |  |  |  |
| q14 |  | 0.699 |  | 0.134 |  |
| q15 | 0.677 |  |  |  |  |
| q16 | -0.121 |  | 0.753 |  |  |
| q17 |  | 0.291 | 0.571 | -0.153 |  |
| q18 |  | 0.179 | 0.62 | 0.102 |  |
| q19 |  | -0.157 | 0.667 |  |  |
| q20 |  |  | 0.238 | 0.614 |  |
| q21 |  | -0.181 |  | 0.859 | 0.248 |
| q22 | 0.202 |  | 0.168 |  |  |
| q23 | 0.344 | -0.112 | 0.545 |  |  |
| q24 | 0.107 | 0.439 |  |  | 0.144 |
| q25 |  | 0.489 |  | 0.582 |  |
| q26 | 0.379 | 0.279 | 0.152 |  | -0.128 |
| q27 | 0.457 |  |  | 0.241 |  |

e. 6-factor structure factor loadings, cumulative variance 41.0%

|  | factor1 | factor3 | factor5 | factor4 | factor2 | factor6 |
| --- | --- | --- | --- | --- | --- | --- |
| q1 |  |  |  |  | 0.417 | 0.263 |
| q2 | -0.115 |  |  | 0.128 | 0.855 |  |
| q3 | -0.110 |  | 0.432 |  | 0.305 | 0.148 |
| q4 |  | 0.157 | 0.182 | -0.103 |  | 0.171 |
| q5 |  |  | 0.201 |  | 0.603 | -0.131 |
| q6 | 0.742 |  |  |  | -0.192 |  |
| q7 |  | 0.132 | 0.229 |  |  |  |
| q8 | 0.371 |  | 0.103 | -0.135 | 0.146 | 0.122 |
| q9 | 0.187 |  | 0.42 |  |  |  |
| q10 | 0.484 |  | -0.218 | -0.144 | 0.155 | 0.168 |
| q11 | 0.588 |  | 0.174 |  |  | -0.215 |
| q12 | 0.534 | -0.272 | 0.282 |  | -0.106 | 0.160 |
| q13 |  |  | 0.798 | -0.123 |  | -0.158 |
| q14 |  |  | 0.623 | 0.127 |  | 0.118 |
| q15 | 0.621 |  |  |  |  | 0.142 |
| q16 | -0.132 | 0.742 |  |  |  |  |
| q17 |  | 0.566 | 0.184 | -0.140 |  | 0.147 |
| q18 |  | 0.613 | 0.131 | 0.103 |  |  |
| q19 |  | 0.661 | -0.101 |  |  |  |
| q20 |  | 0.240 |  | 0.613 |  |  |
| q21 |  |  | -0.171 | 0.837 | 0.244 |  |
| q22 | 0.224 | 0.173 | 0.208 |  |  | -0.157 |
| q23 | 0.318 | 0.532 |  |  |  |  |
| q24 |  |  | 0.158 |  | 0.165 | 0.408 |
| q25 | -0.107 |  | 0.188 | 0.645 |  | 0.421 |
| q26 | 0.303 | 0.135 | -0.119 | 0.127 | -0.133 | 0.611 |
| q27 | 0.423 |  |  | 0.240 |  | 0.111 |
